# Post-exercise rehydration: Comparing the efficacy of three commercial oral rehydration solutions

**DOI:** 10.3389/fspor.2023.1158167

**Published:** 2023-04-27

**Authors:** Donald L. Peden, Mark P. Funnell, Kirsty M. Reynolds, Robert W. Kenefick, Samuel N. Cheuvront, Stephen A. Mears, Lewis J. James

**Affiliations:** ^1^ School of Sport, Exercise and Health Sciences, Loughborough University, Loughborough, United Kingdom; ^2^ Entrinsic Bioscience, LLC, Norwood, MA, United States; ^3^ Sports Science Synergy, LLC, Franklin, MA, United States

**Keywords:** fluid balance, electrolyte balance, sodium, potassium, chloride, drinking, hydration

## Abstract

**Introduction:**

This study compared the efficacy of three commercial oral rehydration solutions (ORS) for restoring fluid and electrolyte balance, after exercise-induced dehydration.

**Method:**

Healthy, active participants (*N* = 20; ♀ = 3; age ∼27 y, $\dot{V}$O_2_peak ∼52 ml/kg/min) completed three randomised, counterbalanced trials whereby intermittent exercise in the heat (∼36°C, ∼50% humidity) induced ∼2.5% dehydration. Subsequently, participants rehydrated (125% fluid loss in four equal aliquots at 0, 1, 2, 3 h) with a glucose-based (G-ORS), sugar-free (Z-ORS) or amino acid-based sugar-free (AA-ORS) ORS of varying electrolyte composition. Urine output was measured hourly and capillary blood samples collected pre-exercise, 0, 2 and 5 h post-exercise. Sodium, potassium, and chloride concentrations in urine, sweat, and blood were determined.

**Results:**

Net fluid balance peaked at 4 h and was greater in AA-ORS (141 ± 155 ml) and G-ORS (101 ± 195 ml) than Z-ORS (−47 ± 208 ml; *P* ≤ 0.010). Only AA-ORS achieved positive sodium and chloride balance post-exercise, which were greater for AA-ORS than G-ORS and Z-ORS (*P* ≤ 0.006), as well as for G-ORS than Z-ORS (*P* ≤ 0.007) from 1 to 5 h.

**Conclusion:**

when provided in a volume equivalent to 125% of exercise-induced fluid loss, AA-ORS produced comparable/superior fluid balance and superior sodium/chloride balance responses to popular glucose-based and sugar-free ORS.

## 1. Introduction

Exercise, particularly in a warm/hot environment, can dramatically increase sweat production to facilitate evaporative cooling and help maintain body temperature. Typically, fluid intake during exercise does not keep pace with fluid lost through sweating (1, 2), meaning dehydration is commonplace at the end of exercise sessions. Dehydration can negatively impact endurance (3, 4), strength (5) and cognitive (6) performance, meaning full recovery of body water lost in one session is important for performance in a subsequent exercise session (7). Given athletes often train more than once a day, this necessitates rapid rehydration in athletes that end exercise dehydrated (7, 8). Additionally, even when a second training session is not undertaken on the same day, the fact that dehydration can negatively affect cognitive performance (6) and/or mood (9), means rapid rehydration might benefit general well-being, as well as performance of other daily activities, such as work, study or even driving.

For complete and rapid rehydration between exercise bouts the most important nutritional factor is beverage volume, with research demonstrating the volume ingested must be more than that lost to account for ongoing fluid losses in the post-exercise period (10, 11). Secondary to volume, is beverage sodium concentration, which has consistently been demonstrated to enhance rehydration (11–14), with sodium concentrations of 40–100 mmol/L reported to maximise post-exercise rehydration. This concentration aligns with typical average human sweat sodium concentrations (15), with some evidence suggesting recovery of sodium balance is required to facilitate recovery of fluid balance (12, 13). However, lower sodium concentrations (∼20–30 mmol/L) produce less consistent benefits to rehydration (12–18); a key consideration for rehydration strategies, given most commercially-available sports beverages contain a sodium concentration of ∼20 mmol/L sodium.

Furthermore, manipulating the carbohydrate and protein/amino acid composition of a beverage might enhance post-exercise rehydration (7). Previous research has suggested that large amounts of carbohydrate must be added to rehydration beverages to enhance rehydration (16, 19, 20). These studies suggest that when other compositional factors are controlled (i.e., sodium concentration), a beverage carbohydrate concentration of >10% is needed to measurably increase rehydration compared to carbohydrate-free/low carbohydrate beverages. The addition of smaller (∼2%) amounts of protein, has been shown in some (21–24) but not all (25–27) studies to increase rehydration, with the source of protein and/or the amino acid composition possibly explaining divergent responses. These findings have important implications for recovery, as in many exercise settings where post-exercise rehydration is required, post-exercise carbohydrate and/or amino acid ingestion might be required to facilitate glycogen resynthesis and/or protein remodeling, respectively (28).

Exercise-induced dehydration without fluid replacement produces hypertonic hypovolaemia or intracellular dehydration (29). This occurs because sweat is typically hypotonic relative to blood (15), raising extracellular osmolality and producing an osmotic gradient that draws water from the intracellular to the extracellular space (29). Another major cause of dehydration in humans is illness that causes diarrhoea and/or vomiting, including infections of the gastrointestinal (GI) tract by bacterium, virus or parasite (30). In such situations, large amounts of electrolytes (particularly sodium and chloride) are secreted into the GI tract and lost in diarrhoea and/or vomit (31). This produces water losses that are typically isotonic compared to blood (30), meaning, extracellular osmolality does not change, little or no water is drawn from the intracellular fluid and the majority of the dehydration is absorbed by the extracellular fluid [termed extracellular dehydration (29)]. For such situations, specific commercially produced oral rehydration solutions (ORS) have been developed, mostly based on World Health Organisation guidelines and evidence from clinical trials (32). ORS have provided an effective alternative to intravenous therapy in cases of dehydration (33), resulting in significantly reduced mortality with diarrheal illnesses (34).

It is well established that glucose can facilitate water and sodium absorption across the intestine through the cotransporter protein SLC5A1 (35). A variety of amino acid-sodium cotransporters also exist among the twenty canonical dietary amino acids, some with similar or superior sodium stoichiometry to glucose (36, 37). Amino acid-sodium cotransporters, SLC38, found in all cells of the body (36), and B0AT1, are shown to stimulate intestinal sodium absorption, absent in the presence of glucose, through the absorption of amino acids (37). Use of amino acids, or food derivatives containing amino acids, in ORS formulations to partially replace glucose has been shown to result in greater net fluid absorption compared to an ORS containing glucose ex-vivo (38) and may enhance rehydration relative to a carbohydrate-electrolyte drink post-exercise (39). Given that glucose may not be ideal in all rehydration situations, the notion that including amino acids in an ORS can help facilitate intestinal water and sodium absorption means the rehydration efficacy of such beverages warrants further investigation.

Interestingly, recommended ORS sodium content (∼75 mmol/L) is consistent with beverages shown to optimise post-exercise rehydration (12, 13). As such, commercial ORS conforming to these guidelines might provide an easy-to-use readily available option to maximise post-exercise rehydration for athletes/exercisers. However, different ORS have different compositions with differing amounts of electrolytes (sodium, potassium, chloride), as well as differing carbohydrate and amino acids contents, which may influence the efficacy of rehydrating post-exercise using these beverages. Whilst some research has compared the rehydration efficacy of different commercially available sports beverages [e.g., Shirreffs et al. 2010 (17)] after exercise-induced dehydration, no research has compared the efficacy of different commercial ORS formulations on post-exercise rehydration, or rehydration in any setting for that matter.

Therefore, the purpose of the present study was to compare the post-exercise rehydration efficacy of a novel amino acid-based ORS developed using HydroActive Technology™ (Entrinsic Bioscience, LLC, Norwood, MA, USA) (AA-ORS) with two established ORS formulations; Pedialyte Classic® (G-ORS; a glucose-based ORS) and Pedialyte Zero® (Z-ORS; a sugar-free ORS). On the basis of superior beverage hydration index outcomes when comparing AA-ORS to G-ORS and other sugar-based commercial beverages in well-hydrated people (40), we hypothesised that AA-ORS would result in greater fluid balance measures (reduced urine output, as well as increased drink retention and net fluid balance) and sodium/chloride balance than both G-ORS and Z-ORS, as well as greater potassium balance compared to Z-ORS in volunteers dehydrated by exercise-heat stress.

## 2. Materials and methods

### 2.1. Ethics approval

All experimental procedures were approved by the Loughborough University Ethics Approvals Human Participants Sub-Committee (LEON 6013). Participants were fully informed of the risks and discomforts associated with all experimental trials before completing a health screen questionnaire and providing written, informed consent.

### 2.2. Participants

Twenty healthy, non-heat acclimated, recreationally active individuals (*N* = 17 male, *N* = 3 female, age 27 ± 5 y, height 1.76 ± 0.09 m, body mass 74.6 ± 11.7 kg, $\dot{V}$O_2_peak 52 ± 15 ml/kg/min, body fat 13.9 ± 3.7%) participated in this study. Participants did not have a history of renal, haematological, or musculoskeletal abnormalities. All participants were living in the U.K. during the months of the study and confirmed that they had not completed exercise training in hot conditions.

### 2.3. Experimental design

Participants visited the laboratory on 4 occasions; a preliminary trial followed by 3 experimental trials, in a randomised, counterbalanced, cross-over order. All exercise was performed on a cycle ergometer (Lode, Groningen, The Netherlands).

### 2.4. Preliminary visit

During the preliminary visit, body mass (AFW-120 K; Adam Equipment, Milyon Keynes, U.K.), height, and body fat [skinfold thickness at biceps, triceps, subscapular, and suprailiac (41)] were obtained. Subsequently, cycling peak oxygen uptake ($\dot{V}$O_2_peak) and peak power output (Wpeak) were determined using an incremental step test to exhaustion. Exercise began at a freely chosen pedal cadence at a workload of 95 W and increased by 35 W every 3 min until volitional exhaustion, despite strong verbal encouragement. Expired gases were collected into a Douglas bag for the final 45–60 s to determine $\dot{V}$O_2_peak. Expired gas was analysed for O_2_ and CO_2_ content (Servomex 1,400 Gas Analyzer; Servomex), volume (Harvard Dry Gas Meter; Harvard Apparatus, Holliston, MA) and temperature (RS Pro digital thermometer; RS Components, Corby, UK).

### 2.5. Pre-trial standardisation

Participants arrived at the laboratory in the morning (time standardised within participant to limit the effects of circadian variation). Participants consumed a standardised breakfast 1.5 h before arrival, consisting of cereal bars totalling 1 g/kg body mass carbohydrate and orange juice mixed with water to provide 0.5 g/kg body mass carbohydrate in a total of 500 ml fluid. The food and fluid were provided to ensure adequate energy availability and pre-trial hydration in anticipation of the exercise protocol. In the 24 h prior to the first experimental trial, participants completed a weighed diet record (food and fluid) using food scales accurate to 1 g and consumed a minimum fluid intake of 40 ml/kg body mass, replicating this food and fluid intake in the 24 h before subsequent trials. Additionally, participants refrained from strenuous physical activity in the 48 h before trials and refrained from alcohol intake in the 24 h before trials. For the measurement of body core temperature during trials, participants ingested a radio-telemetry pill (e-Celcius Performance, BodyCap, Hérouville Saint Clair, France) ∼10–12 h before arrival (i.e., before bed the night before trials). Adherence to all pre-trial requirements was verbally confirmed before each trial.

### 2.6. Experimental protocol

On arrival at the laboratory, and after 10 min seated rest, a small incision was made in a fingertip using a lancet and a 150 uL capillary blood sample was collected into a heparinised capillary tube (Multicap-S; Siemens Healthineers, Erlangen, Germany). Sweat patches (Tegaderm Plus; 3M Health-care, Loughborough, U.K.) were affixed to the right forearm and right scapula (42, 43), after the skin was thoroughly cleaned with distilled water and dried with gauze. Subsequently, gastrointestinal (GI) comfort, thirst sensation [both 0–10 scales (44)] and GI temperature were recorded and participants voided their bladder into a plastic container, before nude body mass was recorded.

### 2.7. Dehydration phase

Participants were dehydrated by intermittent cycling at ∼50% Wpeak in an environmental chamber maintained at 36.3 ± 0.2°C and 52.5 ± 1.0%, until 2.2% of their pre-trial body mass was lost. The total body mass loss target was 2.5% and it was estimated participants would lose the additional 0.3% in the 30 min post-exercise cool down. Participants cycled for 10 min, followed by 5 min rest in the chamber, during which they towel dried and nude body mass was measured. Rate of body mass loss was used to adjust the duration of the final stage to ensure participant body mass loss was as close to 2.2% as possible. In the final min of each exercise stage, rating of perceived exertion [RPE (45)], thirst sensation and thermal sensation [- 10 to +10 (46)] were recorded. Following the second exercise stage, an additional sweat patch was affixed to the left scapula, after thoroughly cleaning and drying the skin. Once the participant reached the target weight, exercise ceased, a timer was started and sweat patches were removed and aspirated into eppendorf tubes using a sterile 10 ml syringe. Participants were allowed 5 min to shower at a standard shower temperature setting, before assuming a seated position in a temperate environment (Table 1) by 15 min post-exercise. After 10 min seated, a fingertip capillary blood sample was collected, GI comfort and thirst were recorded and, at 30 min post-exercise, participants voided their bladder and nude body mass was measured. This body mass loss was used to calculate total body mass (fluid) loss due to exercise and subsequently, to determine the volume of beverage provided during the rehydration phase.

**Table 1:** Laboratory environmental conditions during exercise and resting periods.

	G-ORS	AA-ORS	Z-ORS
*Exercise*			
Temperature (°C)	36.3 ± 0.2	36.4 ± 0.4	36.4 ± 0.5
Relative humidity (%)	52.9 ± 2.1	52.4 ± 0.9	52.8 ± 1.0
*Resting*			
Temperature (°C)	22.5 ± 0.9	22.7 ± 1.2	22.5 ± 0.9
Relative humidity (%)	35.8 ± 6.6	34.2 ± 5.1	34.0 ± 6.4

### 2.8. Rehydration phase

The rehydration phase lasted 5 h and participants consumed 125% of the total body mass losses during dehydration in four even aliquots at hourly intervals (at 0 h, 1 h, 2 h and 3 h). Participants were required to consume the entire volume of each aliquot within 10 min. Participants rested quietly seated in the laboratory for this 5 h, except for urine collection and nude body mass measurements. In the final 5 min of each hour, ambient temperature and relative humidity, as well as GI comfort and thirst were recorded, before participants voided their bladder and had nude body mass measured on the hour. Following the consumption of the second drink (1 h 10 min), participants were provided with a lunch of cereal bars (1.5 kg/body mass of carbohydrate), which they consumed within 10 min. This food was provided due to the extended duration of the protocol and for greater ecological validity, as it is unlikely athletes would restrict food in the 5 h post-exercise in real-world scenarios. Additional fingertip capillary blood samples were collected after 10 min seated rest at 2 h and 5 h of the rehydration phase.

### 2.9. Oral rehydration solution composition and blinding

The composition of the ORS beverages used are presented in Table 2. ORS beverages were administered in a double-blind manner and served to participants in an opaque drinking bottle. Beverages were supplied by Entrinsic Bioscience in bottles containing a unique code and investigators involved in data collection/analysis for the study remained blinded until all analysis had been completed. The AA-ORS beverage contained a proprietary blend of eight amino acids (valine, aspartic acid, serine, isoleucine, threonine, lysine, glycine, tyrosine).

**Table 2:** Oral rehydration solution (ORS) compositions.

	G-ORS	AA-ORS	Z-ORS
Carbohydrate (g/L)	28	0	3
Amino Acid (mmol/L)	0	57	0
Sodium (mmol/L)	45 ± 0	76 ± 3	31 ± 1
Potassium (mmol/L)	23 ± 0	26 ± 2	12 ± 1
Chloride (mmol/L)	38 ± 1	83 ± 2	24 ± 1

Macronutrient concentrations are manufacturer values. Electrolytes determined as described in Methods.

### 2.10. Sample analysis

A blood gas analyser (RAPIDpoint 500e; Siemens Healthineers, Erlangen, Germany) was used for the immediate determination of blood sodium, potassium, and chloride concentration in capillary blood samples. For all urine samples, mass to the nearest 0.1 g, with 1 g assumed to equal 1 ml (Kern PFB-3000-2, Kern, Balingen, Germany) and specific gravity to the nearest 0.001 (PAL-10S Pocket Refractometer, Atago Co LTD, Tokyo, Japan) were immediately determined and an aliquot was frozen at −80°C for later analysis. ORS, urine and sweat samples were analysed for sodium and potassium concentrations by flame photometry (M410C Flame Photometer, Sherwood Scientific Ltd., Cambridge, U.K.) and chloride concentration by coulometric titration (Sherwood Scientific 926S Chloride meter, Sherwood Scientific Ltd., Cambridge, U.K.). For beverage electrolyte concentrations, a sample of eight different beverages for each of the three beverages were measured, with the mean values for each used in calculations of electrolyte balance. Sweat sodium and potassium (42), as well as sweat chloride (43) concentrations were converted to whole body sweat concentrations using published equations. There were issues with the integrity of sweat patches on the forearm in 17 trials (at least 1 trial from 10 separate participants), on the right scapula in 1 trial (i.e., 1 participant) and from the left scapula in 3 trials (3 separate participants). Therefore, sweat collected from the right scapula was used to calculate whole body sweat electrolyte losses, except in one participant, where the left scapula site was used as the only site with sweat samples from all three trials.

### 2.11. Calculations

Fluid retention at time point ^N^ = 100 - (100 x [CumUO^N^ / (CumDV^N^ + CumFW^N^)]
NFB at time point ^N^ = -(1,000 x SL) + CumDV^N^ + CumFW^N^ - CumUO^N^
Electrolyte balance at time point ^N^ = -SE + CumDE^N^ − CumUE^N^
Time to full fluid balance recovery = (1,000 x SL) / {[(1,000 x SL) - NFB^4h^] / 240}

Key: CumUO is cumulative urine output (mL); CumDV is cumulative drink volume (mL); CumFW (mL) is cumulative food water; NFB is the net fluid balance (mL); SL is the total sweat loss (L) calculated from body mass loss from pre-exercise to 0 h; BM is body mass (kg); SE is sweat electrolyte loss (mmol); CumDE is cumulative drink electrolyte concentration (mmol); CumUE is cumulative urine electrolyte concentration (mmol). Superscript denotes the timepoint of measure.

### 2.12. Statistical analysis

All statistical analyses were completed using SPSS (Version 27, IBM). All data were initially checked for normality of distribution prior to analysis using two-factor or one-factor repeated-measures ANOVA. Where the assumption of sphericity was violated, the Greenhouse-Geisser correction was used. Where significant interaction (two-way ANOVA) or main (one-way ANOVA) effects were observed, differences between trials were explored using post-hoc paired *t*-tests or Wilcoxon Signed-Rank tests, as appropriate. The familywise error rate was controlled using the Holm-Bonferroni correction. Cochran's Q test was utilized to compare differences in hydration recovery between beverages (each participant treated as randomized block). Pairwise comparisons were then made using a permutation test with a False Disovery Rate correction applied to the *P*-values (47, 48). All data are presented as mean ± SD. Significance was accepted at *P *≤ 0.05. The required sample size was estimated using an a-prior sample size calculation (GPower) based on a medium effect for net fluid balance across the three trials (*η*p^2^ = 0.07), an *α* of 0.05 and a *β* of 0.2 and a correlation between repeated measures of 0.66, determined from previous studies from our research group examining the effect of amino acid containing beverage to amino-acid-free beverages (21, 22, 25–27). Using these data, it was estimated 17 participants would be required.

## 3. Results

### 3.1. Laboratory conditions

There were no differences in room temperature or relative humidity (Table 1) between trials during exercise (*P* = 0.378 and *P* = 0.432, respectively) or rest (*P* = 0.487 and *P* = 0.538, respectively).

### 3.2. Dehydration phase

Relevant variables for the dehydration phase of the study are presented in Table 3. During the exercise-induced dehydration phase, there were no differences between trials for mean RPE (*P* = 0.214), mean thermal sensation (*P* = 0.275), mean thirst (*P* = 0.656), mean core body temperature (*P* = 0.106) or the total exercise duration required to produce the 2.2% decrease in body mass (*P* = 0.483). Total body mass loss in kg (*P* = 0.402), % body mass loss (*P* = 0.399) and urine output during the dehydration phase (*P* = 0.582) were also not different between trials. Therefore, the volume of beverage consumed during trials was not different in total (G-ORS, 2,372 ± 406 ml; AA-ORS, 2,407 ± 370 ml; Z-ORS, 2,388 ± 361 ml; *P* = 0.423) or for each aliquot (G-ORS, 593 ± 102 ml; AA-ORS, 602 ± 92 ml; Z-ORS, 597 ± 90 ml; *P* = 0.415). The meal participants consumed at 1 h 10 min was identical and contained a small amount of water amounting to 22 ± 5 ml (meal total weight 164 ± 33 g). Finally, there were no significant differences between trials for total sweat losses of sodium (*P* = 0.360), potassium (*P* = 0.776) or chloride (*P* = 0.140) during exercise.

**Table 3:** Dehydration phase measurements.

	G-ORS	AA-ORS	Z-ORS
Total Exercise Duration (min)	73 ± 10	73 ± 12	71 ± 13
Total body mass Loss (kg)	1.90 ± 0.33	1.92 ± 0.29	1.91 ± 0.29
Total body mass Loss (%)	2.56 ± 0.11	2.59 ± 0.09	2.57 ± 0.12
Urine Loss (ml)	68 ± 57	65 ± 45	78 ± 74
Mean exercise GI temperature (°C)	38.1 ± 0.4	37.9 ± 0.5	38.0 ± 0.3
Sweat Sodium Loss (mmol)	49 ± 12	51 ± 15	53 ± 17
Sweat Potassium Loss (mmol)	3.9 ± 0.6	3.9 ± 0.5	3.9 ± 0.5
Sweat Chloride Loss (mmol)	61 ± 13	63 ± 15	66 ± 16
Mean Exercise Thirst	6 ± 2	6 ± 2	6 ± 2
Mean Exercise TS	5 ± 1	5 ± 1	5 ± 1
Mean Exercise RPE	14 ± 2	14 ± 1	14 ± 1

G-ORS; †, G-ORS greater vs. Z-ORS; *P *< 0.05.

### 3.3. Subjective measures

**Figure 1:**
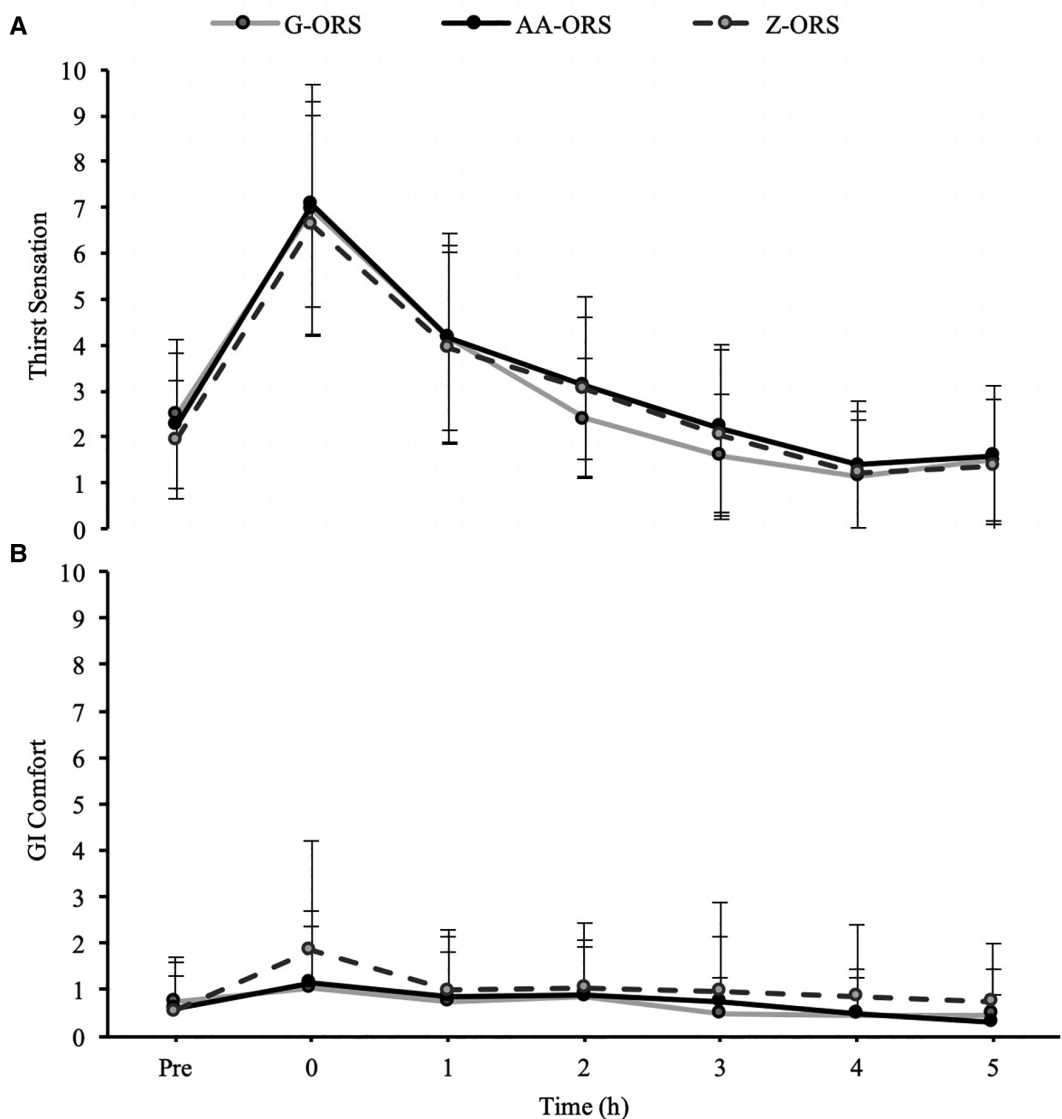
Subjective sensation of (**A**), thirst and (**B**) gastrointestinal (GI) comfort at baseline (Pre) and during the rehydration phase.

There were no interaction effects for thirst (*P* = 0.214; Figure 1A) or GI comfort (*P* = 0.580; Figure 1B), although thirst did change over time (*P* < 0.001), increasing following the dehydration phase before decreasing over the rehydration phase.

### 3.4. Urine output, fluid retention and electrolyte losses

**Figure 2:**
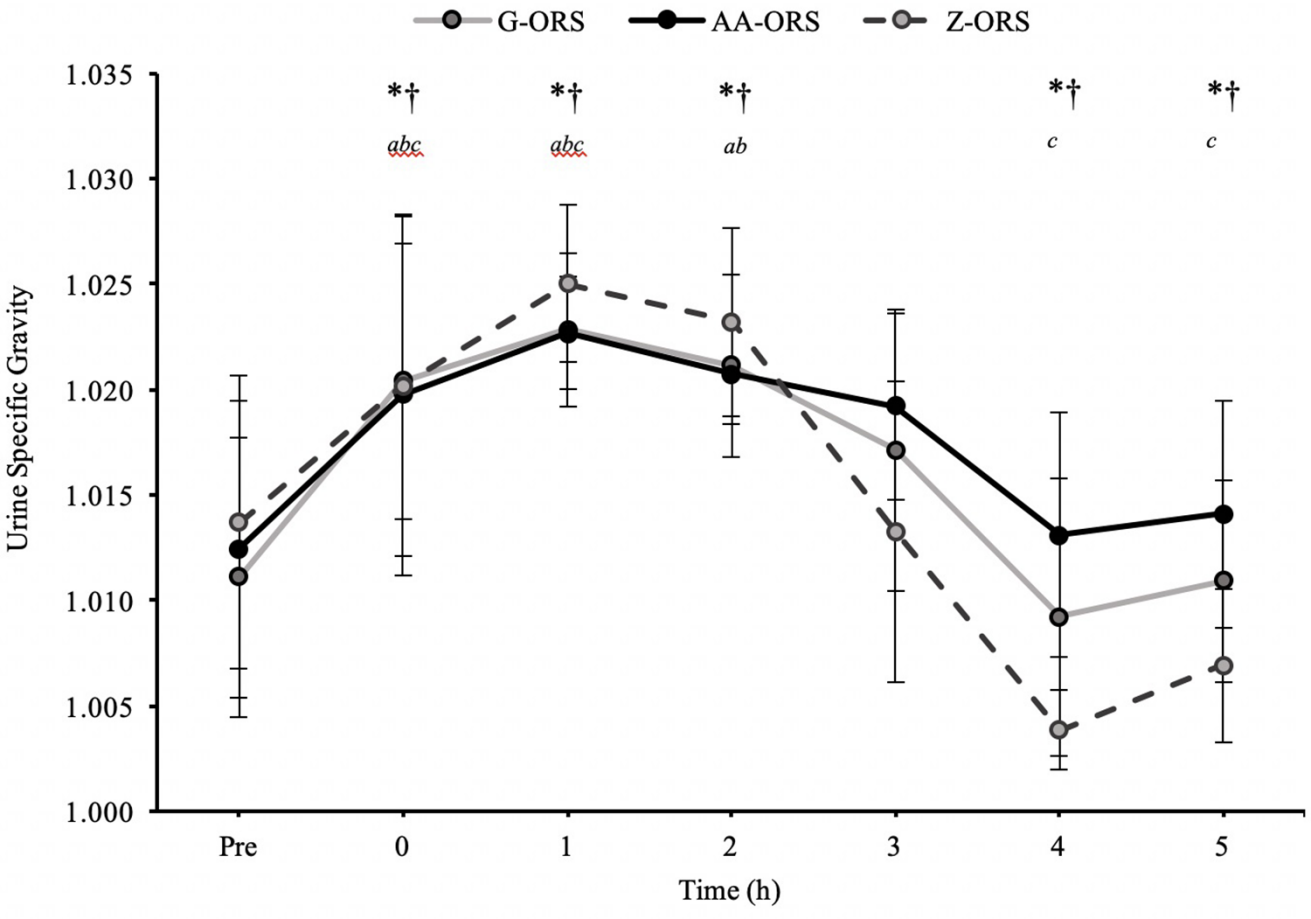
Urine specific gravity from baseline (Pre) during the rehydration phase. *, AA-ORS greater vs. Z-ORS *(P *< 0.05); †, G-ORS greater vs. Z-ORS *(P *< 0.05); *^a^*, G-ORS different vs. baseline, *^b^*, AA-ORS different vs. baseline; *^c^*, Z-ORS different vs. baseline.

There was a time x trial interaction effect (*P* < 0.001) for urine output, with less urine output in AA-ORS and G-ORS compared with Z-ORS at 4 h (*P* = 0.003 and *P* = 0.003, respectively) and 5 h (*P* < 0.001 and *P* = 0.001, respectively; Table 4). There were no significant differences between AA-ORS and G-ORS for urine output (*P* ≥ 0.140). There was a time x trial interaction effect for urine specific gravity (*P* < 0.001), which increased following dehydration in all trials and remained elevated until 3 h in G-ORS (*P* ≤ 0.024) and AA-ORS (*P* ≤ 0.018), and until 2 h in Z-ORS (*P* ≤ 0.018). Additionally, urine specific gravity was lower than baseline at 4 h and 5 h in Z-ORS (*P* ≤ 0.004; Figure 2). Finally, urine specific gravity was lower in Z-ORS compared to AA-ORS at 3–5 h (P < 0.001) and compared to G-ORS at 4–5 h (*P* ≤ 0.005). There was a time x trial interaction effect (*P* < 0.001) for fluid retention (Table 4), with significantly greater retention in AA-ORS and G-ORS than in Z-ORS at 4 h (*P* = 0.003 and *P* = 0.003, respectively) and 5 h (*P* < 0.001 and *P* = 0.001, respectively). There were interaction effects for cumulative sodium (*P* < 0.001), potassium (*P* = 0.002) and chloride (*P* < 0.001) losses in urine (Table 4). Cumulative urine sodium and chloride losses were both greater in AA-ORS compared to Z-ORS from 2 to 5 h (*P* ≤ 0.014) and in AA-ORS compared to G-ORS from 3 to 5 h (*P* ≤ 0.006), with no differences between G-ORS and Z-ORS (*P* ≥ 0.064). Cumulative potassium losses were greater in G-ORS compared to Z-ORS at 1 h, 2 h, 4 h and 5 h (*P* ≤ 0.039) and greater in AA-ORS compared to Z-ORS from 3 to 5 h (*P* ≤ 0.030).

**Table 4:** Cumulative urine output, fluid retention and urine electrolyte losses.

	1 h	2 h	3 h	4 h	5 h
Cumulative Urine Output (mL)					
G-ORS	47 ± 23	99 ± 47	185 ± 88	393 ± 196^c^	542 ± 213^c^
AA-ORS	50 ± 21	122 ± 34	199 ± 52	364 ± 129^a^	485 ± 175^a^
Z-ORS	41 ± 24	85 ± 49	246 ± 128	560 ± 218	779 ± 233
Fluid Retained (%)					
G-ORS	92 ± 4	92 ± 4	90 ± 5	83 ± 8^c^	77 ± 9^c^
AA-ORS	92 ± 3	90 ± 3	89 ± 3	85 ± 6^a^	80 ± 8^a^
Z-ORS	93 ± 4	93 ± 4	86 ± 8	76 ± 10	67 ± 11
Cumulative Urine Sodium Losses (mmol)					
G-ORS	3.6 ± 3.5	7.4 ± 5.6	10.7 ± 6.4	17.4 ± 8.3	25.4 ± 10.1
AA-ORS	4.8 ± 3.1	11.8 ± 6.4^a^	18.0 ± 7.7^ab^	32.0 ± 14.0^ab^	43.5 ± 18.7^ab^
Z-ORS	3.5 ± 2.9	6.7 ± 5.4	11.5 ± 6.9	15.9 ± 8.0	21.6 ± 10.0
Cumulative Urine Potassium Losses (mmol)					
G-ORS	8.5 ± 4.3^c^	19.2 ± 9.2^c^	29.2 ± 12.7	43.2 ± 17.7^c^	55.5 ± 18.6^c^
AA-ORS	8.2 ± 4.0	19.8 ± 8.2	30.7 ± 11.1^a^	47.2 ± 15.4^a^	59.9 ± 18.8^a^
Z-ORS	6.3 ± 4.0	14.0 ± 8.1	24.3 ± 8.8	34.8 ± 12.1	43.6 ± 13.1
Cumulative Urine Chloride Losses (mmol)					
G-ORS	7.2 ± 5.1	15.9 ± 8.9	22.5 ± 11.0	31.2 ± 13.2	41.3 ± 13.6
AA-ORS	8.5 ± 4.8	21.9 ± 9.9^a^	35.0 ± 12.2^ab^	58.3 ± 18.0^ab^	75.8 ± 21.7^ab^
Z-ORS	6.3 ± 5.3	13.1 ± 10.9	21.7 ± 13.0	27.7 ± 14.5	34.0 ± 15.4

^a^ AA-ORS greater vs. Z-ORS.

^b^ AA-ORS greater vs. G-ORS.

^c^ G-ORS greater vs. Z-ORS.

*P* < 0.05.

### 3.5. Fluid balance

**Figure 3:**
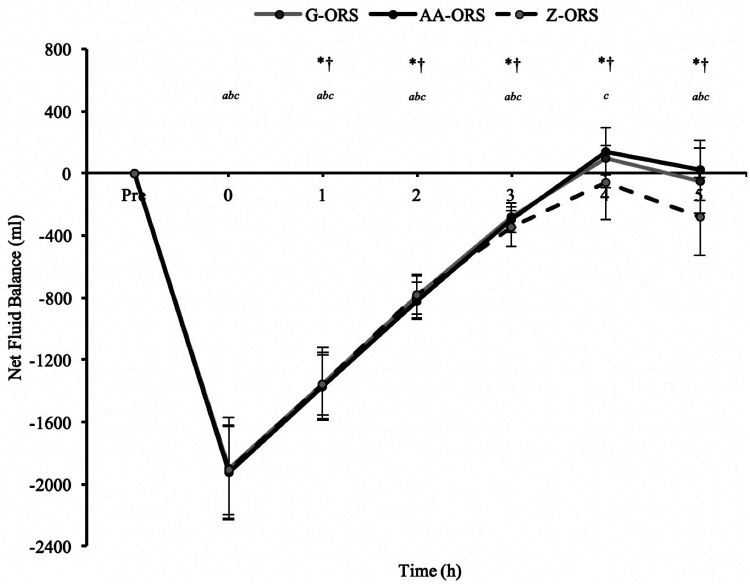
Calculated time to reach full recovery of net fluid balance. *, AA-ORS faster vs. Z-ORS *(P *< 0.05); †, G-ORS faster vs. Z-ORS *(P *< 0.05).

For net fluid balance (calculated from body mass loss during exercise, urine output and beverage intake; Figure 3) there was a significant time x trial interaction effect (*P *< 0.001). Net fluid balance was negative until 3 h in all trials (*P *< 0.001), whereas at 4 h it was not different from baseline in G-ORS and Z-ORS (*P *≥ 0.230), but greater than baseline in AA-ORS (*P *= 0.004). At 5 h, net fluid balance was not different from baseline in AA-ORS and G-ORS (*P *≥ 0.999), but lower than baseline in Z-ORS (*P *< 0.001). Net fluid balance was greater in both AA-ORS and G-ORS compared to Z-ORS from 4 to 5 h *(P *≤ 0.015).

**Figure 4:**
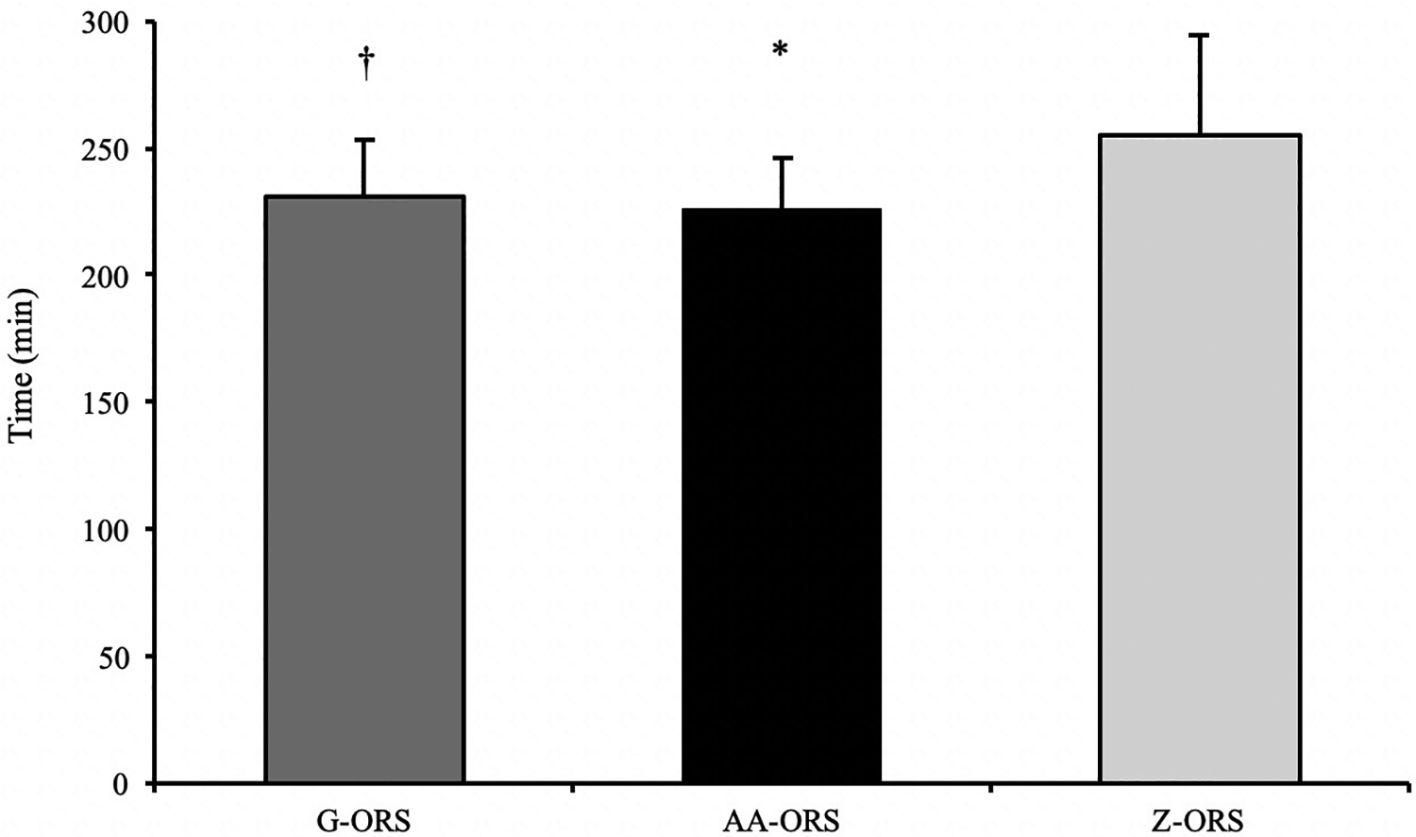
Fluid balance data from baseline (Pre) and during the rehydration phase. *P *< 0.05: *, AA-ORS greater vs. Z-ORS; †, G-ORS greater vs. Z-ORS; *^a^*, G-ORS different vs. baseline, *^b^*, AA-ORS different vs. baseline; *^c^*, Z-ORS different vs. baseline.

**Figure 5:**
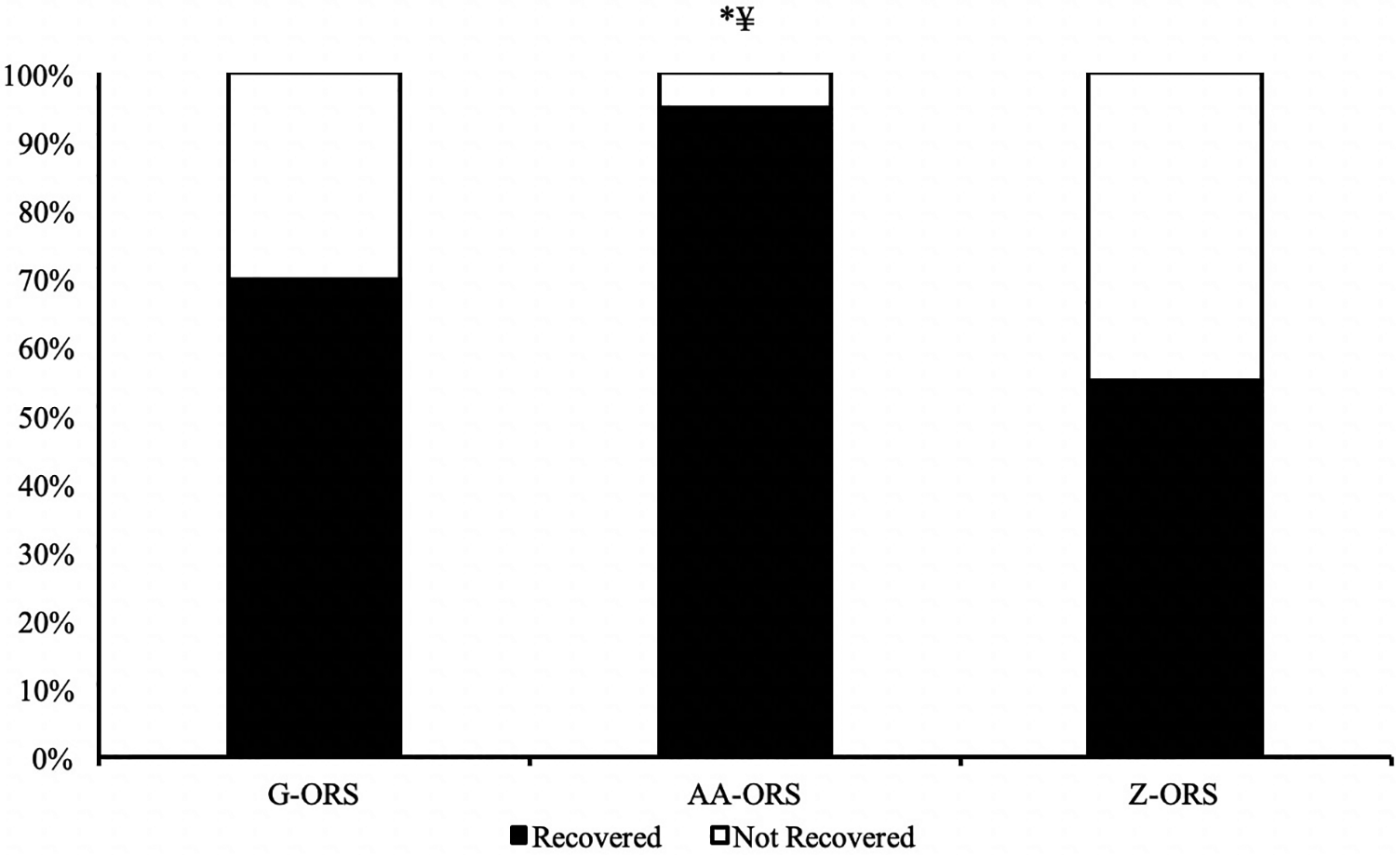
Percentage of participants fully reaching full restoration of fluid balance. *, AA-ORS greater vs. Z-ORS *(P *< 0.05); ¥, AA-ORS greater vs. G-ORS *(P *< 0.05).

The calculated time at which participants reached full recovery of net fluid balance (Figure 4) differed between trials (*P *< 0.001), with faster restoration in AA-ORS (*P *< 0.001) and G-ORS (*P *< 0.001) compared with Z-ORS. There was no difference in the calculated time for restoration between AA-ORS and G-ORS (*P *= 0.370). For the number of participants reaching full recovery of fluid balance (i.e., NFB ≥ 0), Cochran's Q test revealed a significant effect of beverage (*χ*^2 = 10.889, *P* = 0.004; Figure 5). Comparisons between individual trials revealed a greater percentage of participants completely restoring fluid balance in AA-ORS compared with Z-ORS (z = 2.83, *P* = 0.014), or G-ORS (z = −2.24, *P* = 0.038). There was no difference between G-ORS and Z-ORS (z = 1.34, *P* = 0.18).

### 3.6. Electrolyte balance

**Figure 6:**
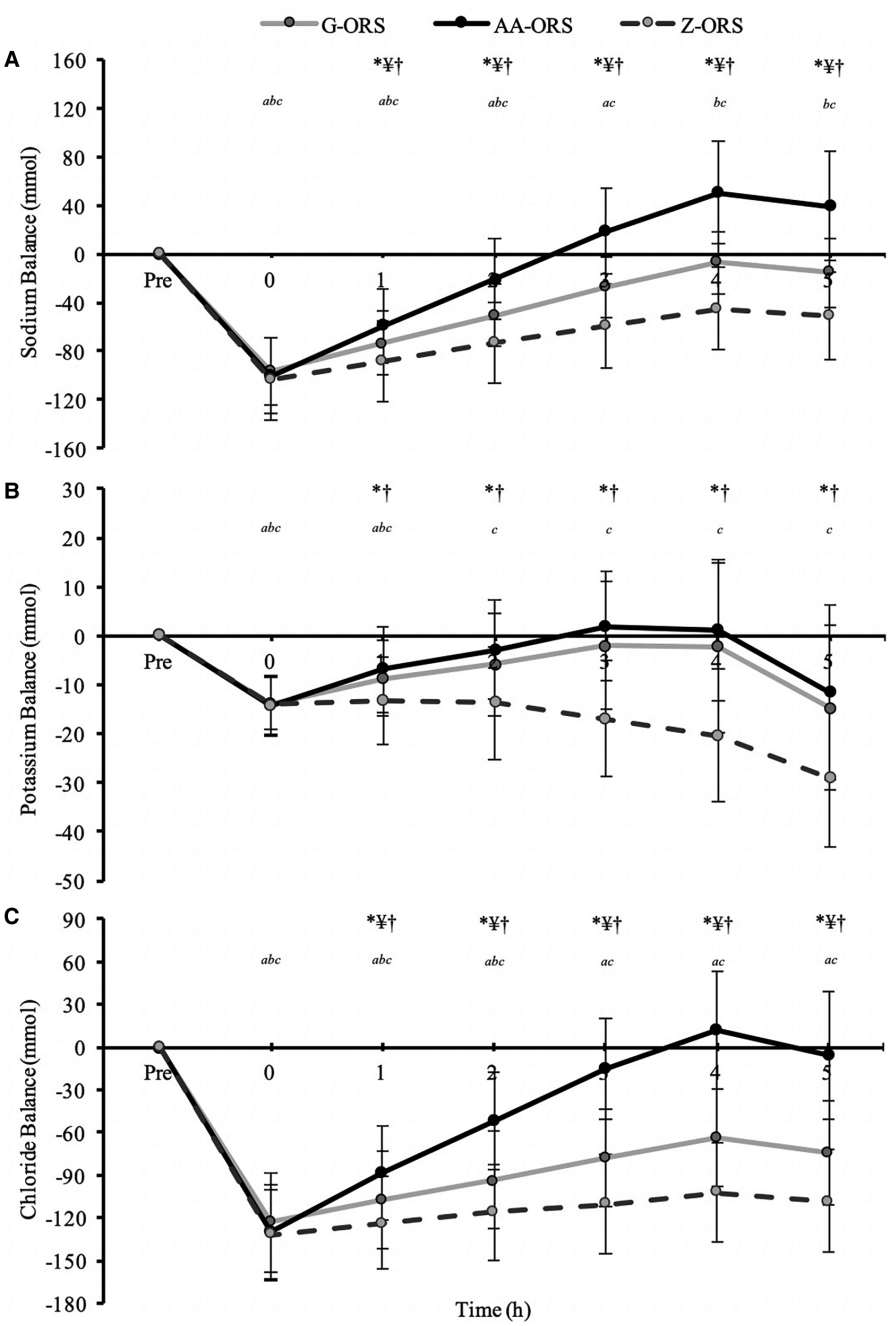
Electrolyte balance data from baseline (Pre) and during the rehydration phase. (**A**) Sodium; (**B**)*,* Potassium; (**C**), Chloride. *P *< 0.05: *, AA-ORS greater vs. Z-ORS; ¥, AA-ORS greater vs. G-ORS; †, G-ORS greater vs. Z-ORS; *^a^*, G-ORS different vs. baseline, *^b^*, AA-ORS different vs. baseline; *^c^*, Z-ORS different vs. baseline.

There were time x trial interaction *(P *< 0.001) effects for sodium, potassium, and chloride balance (Figure 6). The exercise bout produced a negative balance of all three electrolytes (Table 3; *P *< 0.001), which was not different between trials for sodium *(P *≥ 0.579), potassium *(P *> 0.999) and chloride *(P *≥ 0.290). Post-exercise, sodium balance (Figure 6A) was reduced compared to baseline at all time points in all trials *(P *≤ 0.001), except in AA-ORS at 2–3 h *(P *≥ 0.067) and in G-ORS at 4–5 h *(P *≥ 0.082), where it was not different from baseline, and at 4–5 h in AA-ORS *(P *≤ 0.004), where it was greater than baseline. Sodium balance was greater in AA-ORS compared to both G-ORS and Z-ORS, and in G-ORS compared to Z-ORS from 1 to 5 h *(P *≤ 0.031). Post-exercise, potassium balance (Figure 6B) was reduced compared to baseline at all time points in all trials *(P *≤ 0.017), except at 2–5 h in AA-ORS *(P *≥ 0.097) and 2–4 h in G-ORS *(P *≥ 0.071), where there were no differences compared to baseline. Potassium balance was greater in AA-ORS compared to Z-ORS from 1 to 5 h *(P *≤ 0.012). Post-exercise, chloride balance (Figure 6C) was reduced compared to baseline at all time points in all trials *(P *≤ 0.020), except at 3–5 h in AA-ORS *(P *≥ 0.305), where there were no differences from baseline. Chloride balance was greater in AA-ORS compared to both G-ORS and Z-ORS, and in G-ORS compared to Z-ORS from 1 to 5 h *(P *≤ 0.012).

### 3.7. Blood electrolyte concentrations

**Figure 7:**
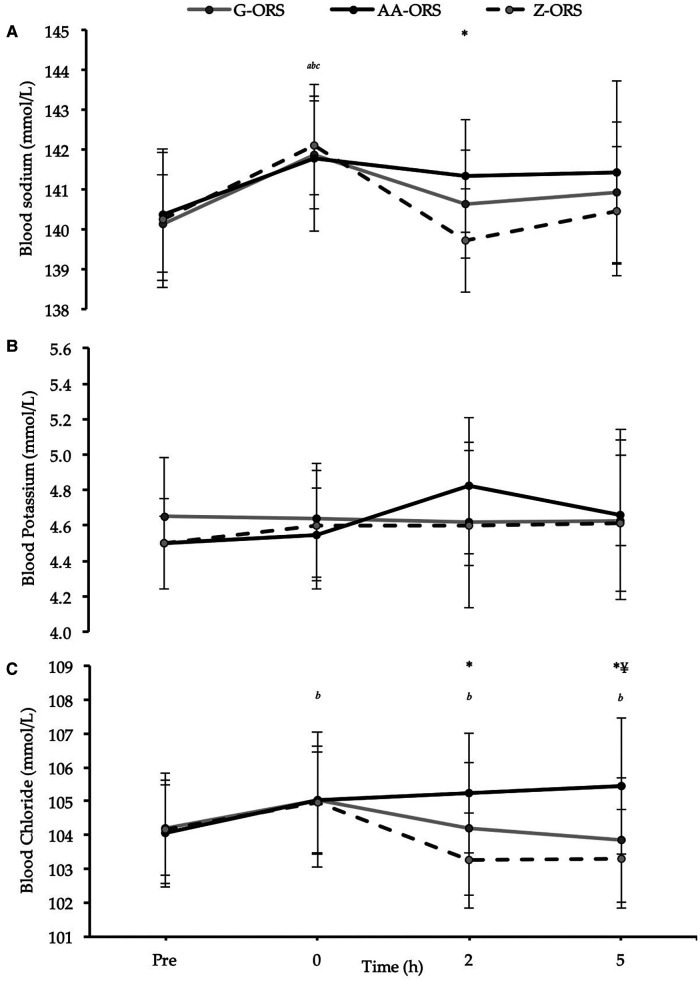
Blood electrolyte concentrations from baseline (Pre) and during the rehydration phase. (**A**) Sodium; (**B**) Potassium; (**C**) Chloride. *P *< 0.05: *, AA-ORS greater vs. Z-ORS; ¥, AA-ORS greater vs. G-ORS; ^a^, G-ORS vs. baseline, ^b^, AA-ORS different vs. baseline; ^c^, Z-ORS different vs. baseline.

There were time x trial interaction effects *(P *≤ 0.019) for blood sodium, potassium, and chloride concentrations (Figure 7). Post-exercise blood sodium concentrations were elevated *(P ≤ *0.001) compared with baseline but had returned to baseline at 2 and 5 h *(P *≥ 0.232) in all trials (Figure 7A). Blood sodium concentration was greater in AA-ORS compared with Z-ORS at 2 h *(P *= 0.003). There were no differences between trials at any time point for blood potassium concentration *(P *≥ 0.136), and compared with baseline, there were no differences in any post-exercise blood potassium concentrations *(P *≥ 0.256), except an increase at 2 h in AA-ORS *(P *= 0.003; Figure 7B). Compared to baseline, post-exercise blood chloride concentration was elevated in AA-ORS *(P = *0.004) and remained elevated at 2 and 5 h *(P *≤ 0.007), with no differences to baseline at any time point in G-ORS or Z-ORS *(P *≥ 0.076; Figure 7C). Blood chloride concentration was greater in AA-ORS compared with Z-ORS at 2 h *(P *< 0.001) and compared to G-ORS and Z-ORS at 5 h *(P *≤ 0.021).

## 4. Discussion

The present study demonstrates that following exercise-induced dehydration of ∼2.6% body mass, all fluid balance markers (i.e., urine output, fluid retention and body mass change), were enhanced with AA-ORS and G-ORS compared to Z-ORS, without any significant differences between AA-ORS and G-ORS. Furthermore, sodium and chloride balance were significantly greater in AA-ORS compared to both G-ORS and Z-ORS, as well as in G-ORS compared to Z-ORS from 1 h post-exercise until the end of the study (i.e., 5 h post-exercise). Potassium balance was significantly greater in AA-ORS and G-ORS compared to Z-ORS from 1 to 5 h post-exercise, with no difference between AA-ORS and G-ORS. These results are in line with our hypotheses, apart from that fluid balance measures were not different between AA-ORS and G-ORS. These findings suggest that an amino acid-based ORS is a viable alternative to a glucose-based ORS to support post-exercise rehydration, and in terms of sodium and chloride balance, provides superior recovery of electrolyte balance post-exercise.

Several measures of fluid balance were made concurrently during the rehydration phase of the present study to explore potential differences among beverages. As intended, there was no difference between trials for body mass loss (i.e., fluid loss) during the exercise-induced dehydration phase, and, consequently, the volume of drink consumed in the rehydration phase was also not different between trials. Therefore, differences between trials for post-exercise fluid balance variables were caused by differences in physiological responses to the ingested beverages. Total urine output over the 5 h rehydration phase was reduced *(P *< 0.001) in AA-ORS (485 ± 175 ml) and G-ORS (542 ± 213 ml) compared to Z-ORS (779 ± 223 ml), with no difference between AA-ORS and G-ORS *(P *< 0.304). Ultimately, these effects on urine output meant that similar effects were apparent for fluid retention and net fluid balance over the rehydration phase. These fluid balance data are mainly consistent with the results of previous studies that have manipulated the sodium and/or chloride composition of post-exercise rehydration beverages (10–13).

In general, previous studies demonstrate that recovery of fluid balance after exercise-induced dehydration is directly related to the sodium content of the ingested beverage, but it seems that ≥40 mmol/L sodium is generally required to observe significant differences compared to lower sodium concentration beverages (12–14). When comparing Z-ORS to AA-ORS and G-ORS, it is apparent that the increases in sodium and/or chloride concentration in the beverages produced significant increases in fluid balance, which was expected due to these beverages containing >40 mmol/L sodium. The Z-ORS beverage contained amounts of sodium and chloride that often produce equivocal rehydration benefits compared to water or electrolyte-free solutions. Studies examining drinks with sodium concentrations in the range 24–30 mmol/L (i.e., similar to Z-ORS) compared to water or placebo beverages demonstrate no significant benefit to fluid balance measures (12–14, 17, 18, 49–51), with only one exception to our knowledge (52). It is worth noting that in this study, the electrolyte containing beverage also contained 7.6% carbohydrate, but Z-ORS only contained 0.3%.

Data comparing beverages with sodium concentration >50 mmol/L are scarce in the literature and have observed inconsistent findings. Two studies have compared post-exercise beverages with sodium concentrations of ∼100 mmol/ and ∼50 mmol/L (12, 13), with one (13) observing increased fluid balance with ∼100 mmol/L sodium compared to ∼50 mmol/L sodium, but not the other (12). Therefore, it is perhaps unsurprising that the present study found no significant difference between AA-ORS and G-ORS, given that the difference in sodium concentration was not as large as these previous studies (∼31 mmol/L). Furthermore, the Cochran's Q test suggested that the proportion of participants returning to net fluid balance (i.e., ≥0 ml over the rehydration phase) was greater in AA-ORS than Z-ORS and G-ORS (*P *≤ 0.05), with 19 (out of 20) returning to net fluid balance in AA-ORS compared with 11 and 14 in Z-ORS and G-ORS, respectively. This suggests that the AA-ORS beverage might provide a more consistent response across different participants. However, it is important to note that on average the difference between AA-ORS and G-ORS amounted to ∼40 ml at 4 h and ∼67 ml at 5 h and was not significantly different between trials *(P *> 0.999).

The findings for sodium and chloride balance are consistent with previous rehydration experiments that have manipulated sodium and/or chloride concentrations of rehydration beverages (12–14). These findings clearly demonstrate that AA-ORS outperformed both G-ORS and Z-ORS. Increasing the sodium and chloride concentration in post-exercise beverages appears to have little effect on subsequent urinary electrolyte output, up to a beverage sodium chloride concentration of ∼50–60 mmol/L; beyond this concentration, increased electrolyte losses in urine are apparent (12, 13). In the present study, the increase in sodium and chloride concentrations from Z-ORS (sodium 31 mmol/L; chloride 24 mmol/L) to G-ORS (sodium 45 mmol/L; chloride 38 mmol/L) did not influence urinary electrolyte output. However, increasing sodium and chloride levels to those found in AA-ORS (sodium 76 mmol/L; chloride 83 mmol/L) produced a small, but significant increase in total urine sodium (∼20 mmol) and chloride (∼40 mmol) output compared to the other trials. However, these increased losses were not sufficient to mitigate the additional sodium and chloride consumed in the AA-ORS beverage, resulting in greater sodium and chloride balance in this trial.

It is interesting that sodium and chloride balance were greater after the AA-ORS beverage compared to the G-ORS beverage, but that these increases did not elicit alterations in urine output or other fluid balance measures. Similar findings have been reported previously (12). There are several reasons why a more positive sodium and chloride balance could be beneficial, depending on the situation. Firstly, the additional sodium and chloride balance could be of value in retaining subsequently ingested fluid that is likely to be more dilute (e.g., water or similar), thus possibly helping to enhance subsequent fluid balance or maintain homeostasis. Indeed, there was a significant increase in blood chloride concentration at 5 h in AA-ORS compared to the other trials that might assist in this manner. Additionally, it is important to consider that the primary intended use of the beverages in the present study is in situations of isotonic hypovolaemia [i.e., extracellular dehydration (29)], as observed when the dehydration is produced through diarrhoeal disease (30). With diarrhoea and/or vomiting induced dehydration we would expect much greater relative losses of sodium and chloride than following the exercise-induced dehydration used in the present study (29, 39). Although speculative, this means that the increased sodium and chloride balance reported here might become meaningful for dehydration under such conditions and might be expected to facilitate increased rehydration (39), with potentially important implications. This is something future studies should seek to examine.

Potassium balance was greater throughout the rehydration phase in AA-ORS and G-ORS compared to Z-ORS, but the beverage concentrations of potassium were a lot closer than for sodium and chloride. Interestingly, and in contrast to sodium and chloride, the small increase in potassium concentration from that of Z-ORS beverage (∼12 mmol/L) to AA-ORS and G-ORS beverages (∼26 mmol/L and ∼23 mmol/L, respectively), did produce significant increases in potassium excretion in urine over the rehydration phase. The exercise-induced dehydration produced only small losses of potassium in sweat (∼4 mmol/L or ∼7 mmol in total), consistent with previous studies (42, 43). In fact, the potassium losses in urine were similar to that in sweat (∼7 mmol), but total losses (∼14 mmol) were still less than was consumed in rehydration beverages in all trials, meaning it is unlikely any differences in potassium balance explains the differences observed for fluid balance. For the primary intended purpose of the beverages used in the present study (i.e., situations of diarrhoeal disease), the dehydration present would likely also produce greater potassium losses compared to exercise-induced dehydration (30, 39). Indeed, potassium containing beverages used for post-exercise rehydration have produced equivocal results (17, 53–55), with concentrations of 30–50 mmol/L potassium demonstrating no increase in post-exercise rehydration in some studies (17, 54). Therefore, given the ∼11–14 mmol/L difference between beverages in the present study, it seems unlikely that differences in potassium content explain the fluid balance observations.

The finding that AA-ORS was comparable to G-ORS for fluid balance measures and superior for sodium and chloride balance may be of use to athletes looking to recover from dehydration without ingesting glucose or sugars. Whilst it is well established that glucose can facilitate water and sodium absorption across the small intestine through the cotransporter protein SLC5A1 (35), the inclusion of an appropriate mixture of amino acids in AA-ORS may facilitate similar sodium stoichiometry and water carrying benefits to SLC5A1, but with a wider variety of amino acid-co-transporters, such as SLC38 (36) and B0AT1, which stimulate intestinal sodium absorption, absent in the presence of glucose, through the absorption of amino acids (37). From a health perspective, providing amino acids instead of glucose might prevent glucose related complications to dental (56), or metabolic health (57, 58) or even energy balance (28). Some athletes (particularly endurance athletes) may choose to avoid carbohydrate ingestion between consecutive training sessions to facilitate low carbohydrate availability at the second session and enhance endurance exercise adaptations (59). In such situations, post-exercise rehydration may be warranted and therefore athletes using such a training strategy may use an amino acid-based ORS to enhance rehydration without compromising their other training goals. Finally, combat sports athletes, who make weight before competition, typically use extreme dehydration and food restriction (60) to reach the required weight, with some losing > 13% of body mass in the week before weigh-in (61). Given food restriction increases sodium, potassium, and chloride losses (62), recovery of these electrolyte losses (as well as those lost through sweating) will be paramount for effective recovery (63). Clearly, in such cases, greater levels of electrolytes in a rehydration drink may facilitate better recovery, but with the volume of rehydration needed over a protracted rehydration period (24–36 h), a carbohydrate-free ORS may allow the separation of fluid and carbohydrate recovery strategies, as required.

In conclusion, the present study demonstrates that following exercise-induced dehydration, and when consumed in a volume equivalent to 125% of the exercise fluid loss, an amino acid-based ORS (AA-ORS) restores net fluid balance similarly compared with a glucose-based ORS (G-ORS), and superiorly, compared to a sugar-free ORS formulation (Z-ORS), potentially offering a solution to optimise post-exercise rehydration in numerous settings. Furthermore, AA-ORS produced significant increases in recovery of sodium and chloride balance compared to current glucose-based (G-ORS) and sugar-free (Z-ORS) commercial ORS. This may help facilitate longer-term recovery and/or provide the rationale for enhanced recovery in situations where electrolyte losses are proportionally greater (i.e., diarrhoeal disease/extracellular dehydration). Future studies should seek to examine the efficacy of different ORS formulations in such situations.

## Data Availability

The raw data supporting the conclusions of this article will be made available by the authors, without undue reservation.
